# The Use, Perceived Effectiveness and Safety of Herbal Galactagogues During Breastfeeding: A Qualitative Study

**DOI:** 10.3390/ijerph120911050

**Published:** 2015-09-07

**Authors:** Tin Fei Sim, H. Laetitia Hattingh, Jillian Sherriff, Lisa B.G. Tee

**Affiliations:** 1 School of Pharmacy, Curtin University, Perth, WA 6845, Australia; E-Mails: l.hattingh@curtin.edu.au (H.L.H.); l.tee@curtin.edu.au (L.B.G.T.); 2 School of Public Health, Curtin University, Perth, WA 6845, Australia; E-Mail: j.sherriff@curtin.edu.au

**Keywords:** breastfeeding, infant health, herbal medicines, galactagogues, women’s perspectives

## Abstract

The World Health Organization recommends breastfeeding as the normal infant feeding method and that infants being breastfed should be regarded as the control group or norm reference in all instances. There are many factors which could contribute to a new mother ceasing breastfeeding early, with the most commonly reported reason being perceived insufficient breast milk supply. The use of herbal galactagogues is increasingly common worldwide. Literature review identified a need for more research in the area of herbal galactagogue use during breastfeeding. Twenty in-depth semi-structured interviews were undertaken with breastfeeding women who used herbal galactagogues, to document use and explore their perceived effectiveness and safety of herbal galactagogues. Several indicators of breastfeeding adequacy were mentioned as participants described their experiences with the use of herbal galactagogues. Confidence and self-empowerment emerged as an over-arching theme linked to positive experiences with the use of herbal galactagogues. Despite the lack of clinical trial data on the actual increase in measured volume of breast milk production, indicators of breastfeeding adequacy boosted participants’ confidence levels and resulted in psychological benefits. This study highlighted the importance of considering the potential psychological benefits of using herbal galactagogues, and how this translates into breastfeeding adequacy.

## 1. Introduction

The World Health Organization (WHO) recommends breastfeeding as the normal infant feeding method and that infants being breastfed should be regarded as the control group or norm reference in all instances [1]. The Australian Dietary Guidelines published by the National Health and Medical Research Council (NHMRC) provide dietary and nutritional recommendations to promote the overall well-being and health of the Australian population [2]. The *Eat for Health: Infant Feeding Guidelines, Information for Health Workers* was published by the NHMRC in 2012, providing advice on recommended infant feeding practices [2,3]. It is recommended that all infants should be exclusively breastfed until the age of six months, with breast milk being the only source of nutrition for the infant [3]. The recommendation by the NHMRC is in line with advice provided by the American Academy of Paediatrics and World Health Organization (WHO) [4,5].

The percentage of women who choose breastfeeding instead of artificial feeding immediately postpartum has increased from approximately 48% in the 1970s to over 90% in the past decade [6]. Despite the rise in breastfeeding initiation rates over the past decades, duration of breastfeeding remains an area to be improved in Australia [7,8]. Many studies have been conducted to investigate factors affecting breastfeeding practices and reasons for early breastfeeding cessation [9,10]. An understanding of the various factors affecting breastfeeding practices in Australia will assist in addressing reasons and issues that impact on the duration of breastfeeding.

There are many factors which could contribute to a new mother not initiating breastfeeding or to cease breastfeeding early. Several studies have been conducted in Australia to identify women’s reasons for not initiating breastfeeding or ceasing breastfeeding earlier than recommended in the Australian Dietary Guidelines [3,8]. The National Health Survey conducted in 2001 by the Australian Bureau of Statistics [11] revealed that the most commonly self-reported reason for discontinuation of breastfeeding was inadequate breast milk supply (30%), followed by a feeling that it was time to stop (23%), breastfeeding-related problems such as cracked nipples (10%), and having to return to work (8%). Women’s socioeconomic backgrounds also influence their breastfeeding practices. Results of a recent study in Chicago, United States of America (USA), supported the literature in that women from a lower income or socioeconomic group were more likely to discontinue breastfeeding earlier and that the most common reported reasons for discontinuing breastfeeding included: the perception of insufficient milk supply (46%), maternal medical problems (13%), and having to return to school or work (13%) [12]. While deficient mammary gland tissue, maternal hormone imbalance, poor breastfeeding technique or latching leading to ineffectual milk removal can all contribute to low milk supply [4,13], women’s inaccurate perception of insufficient milk supply and the lack of confidence or reassurance have also been shown to affect the duration and success of breastfeeding [14].

Galactagogues are a group of substances or medicines either proven or believed to aid lactation during initiation and maintenance stages, thereby increasing human breast milk supply [13,15,16,17,18,19]. In other words, galactagogues are substances which may be used by women to induce, increase or maintain milk production [19]. Galactagogues are available in Australia in the form of either conventional medicines or of herbal origin. Dopamine D_2_ receptor antagonists, such as metoclopramide [20,21,22,23,24] and domperidone [25,26], are conventional galactagogues that are most commonly used in clinical practice with supported evidence. These agents increase milk supply and production by increasing the levels of prolactin in the maternal plasma [16,22,26].

Throughout the world, women have used many alternative approaches in an attempt to increase milk production such as following special diets and the use of herbal or natural substances. Mothers of different cultural and ethnic backgrounds often choose different approaches according to their tradition or experience [27,28,29,30,31,32]. Herbal medicines commonly believed to aid lactation include fenugreek (*Trigonella foenum-gracum*) [4,33,34,35,36,37], blessed thistle (*Cnicus benedictus*) [4,13,19,38], milk thistle (*Silybum marianum*) [39,40], goat’s rue (*Galega officinalis*) [4,41,42], marshmallow (*Althaea officinalis*) [4], fennel (*Foeniculum vulgare*) [4,13], torbangun (*Coleus amboinicus Lour*) [36], nettle (*Urtica dioica*) [4,13] and black seed (*Nigella sativa*) [43]. Many of these herbal medicines, in particular fenugreek, have gained popularity in the Western world as galactagogues. Fenugreek is the herbal remedy that is most commonly recommended for deficient milk supply and has been listed as L3 (moderately safe) by Hale [33].

Despite their long history of use, scientific evaluation is lacking to confirm the clinical efficacy of most herbal medicines as galactagogues. Many anecdotal reports suggest effectiveness of fenugreek in promoting lactation, including a survey of La Leche League (a breastfeeding organization) leaders and lactation consultants indicating positive effects in milk supply in approximately 75% of lactating women [44]. Detailed protocols in regards to the use of fenugreek were published by Huggins in 1998 [45] and later by Newman and Pitman in 2000 [46]. According to the Academy of Breastfeeding Medicine’s protocol #9 [47], the usual recommended dosage of fenugreek for stimulating lactation is one to four capsules three or four times daily. As there is currently no standardization of fenugreek content across various brands and sources, Hale and Hartmann [4] suggest that calculation of a total daily dose of 1.74 g to 4.9 g may be more practical and useful. On the other hand, the German Commission E recommends the use of fenugreek as a herbal galactagogue at a total dose of 6 g of fenugreek seeds daily in divided doses [48].

The aim of the study was to document the pattern of use, safety and perceived effectiveness of herbal galactagogues during breastfeeding based on breastfeeding women’s personal experiences and observations. Gaining an understanding of their perspectives around why and how they have chosen to use herbal galactagogues over, or in combination with, conventional options, their experiences and the factors or indicators that influenced their breastfeeding adequacy, will provide insight into the potential value of herbal galactagogues and identify research gaps to inform direction of future studies. 

## 2. Materials and Methods

In-depth semi-structured interviews were conducted with women from Perth, Western Australia, who used herbal galactagogues. The Curtin University Human Research Ethics Committee (HREC) approved this study (HR85/2012). All participants provided written consent. The researcher rigorously adhered to an open style of questioning and avoided making comments on participants’ choices. Participants were reassured that their participation was completely voluntary and anonymous and they could withdraw at any time during the study. To be eligible for the study, prospective participants had to be at least 18 years of age, be breastfeeding or have breastfed in the last 12 months, and were users of herbal galactagogues during breastfeeding. All participants were de-identified and codes were used in the analysis. 

### 2.1. Justification of Research Methodology and Sample Size

Qualitative research methodology was chosen for this study as it allows the collection of relevant, rich and in-depth information, enabling researchers to explore the attitudes and perspectives of individuals in the context of the their individual circumstances [49,50,51]. The determination of sample size in qualitative research should be based on the research purpose, judgment and experience [52]. A similar study conducted by Westfall [13] in British Columbia which investigated the use of herbal galactagogues involved a total of 23 participants. The point of data saturation was considered reached when no new themes were emerging. Taking into consideration the aforementioned points, the research team was confident that interviews with 20 participants were adequate to reach saturation of data, at the same time enabling in-depth data analysis. 

### 2.2. Recruitment of Participants

Purposeful chain sampling targeting breastfeeding women who had an interest in herbal medicines and who were using or recently used herbal galactagogues, was used to recruit participants. Participants recruited from local naturopathic clinics, a health centre, two community pharmacies with a focus on naturopathy or through media releases in parenting papers and announcement via the Curtin FM 100.1 Perth radio station, were asked to provide study and contact details to friends or family members who were eligible to participate, and this chain was continued. 

### 2.3. Data Collection and Analysis

All participants were reassured at the start of the interviews that the study was not to advocate or discourage the use of any medicines or choice of therapy, but rather to obtain insight into participants’ experiences and perception. An interview guide (10 closed-ended questions; seven open-ended questions) was used and to ensure the clarity of the questions and that the research objectives were addressed, the interview guide was validated through seeking for colleagues’ feedback and pilot testing on two breastfeeding women. Individual interviews were conducted either face-to-face or via telephone based on participants’ preferences. All interviews were audio-recorded, then manually transcribed verbatim for the purpose of analysis. Participants’ responses to closed-ended questions were analysed using descriptive analysis to summarise the findings. Data collected on the type(s) of herbal galactagogue(s) used, reasons for use, sources of recommendations and information resources, routes and dosage administered, commencement and duration of administration were summarised. Data were systematically separated into categories based on the name or type of the herbal galactagogue(s) discussed. 

The opened-ended questions in the interview guide that explored the experiences and perspectives of participants were analysed using thematic analysis as described by Boyatzis [53]. Qualitative findings of the participants’ general perspectives and attitudes towards the use of herbal medicines during breastfeeding and their views on the accessibility and quality available resources, as well as their perspectives towards the role of health professionals, are reported elsewhere [54].

## 3. Results

Between October 2012 and April 2013, a total of 20 in-depth semi-structured interviews with breastfeeding women were undertaken. All participants (18 of Caucasian descent and two of Asian descent) were users or recently used herbal galactagogues. Interviews were between 18 and 78 min. Of the 20 participants, fifteen were first-time mothers.

### 3.1. Pattern of Use

Data about herbal medicines used were categorised based on the name or type of the herbal galactagogue(s) discussed: (i) fenugreek as a sole ingredient; (ii) fenugreek and blessed thistle as a combination product; and (iii) naturopaths’ own “lactation tincture” with a combination of herbal ingredients. Summaries of patterns of use are provided in Table 1, Table 2 and Table 3, respectively. All 20 participants had used fenugreek either as a sole ingredient or in combination with other herbal ingredients. Ten participants had used fenugreek as a sole ingredient to promote breastfeeding (Table 1), whilst three had used fenugreek and blessed thistle as a combination product (Table 2), and seven used a naturopath’s own “lactation tincture” with a combination of herbal ingredients (Table 3). The most popular formulation or brand of fenugreek was Nature’s Own^®^ Fenugreek 1000 mg, which nine participants had used. Participants commenced herbal galactagogue therapy at various times following delivery of the baby, with one participant commencing as early as three days and one as late as seven months postpartum. Duration of administration varied from one week to nine months.

Participants used herbal galactagogues during breastfeeding for various reasons, including perceived insufficient milk supply (*n* = 9), health professional-diagnosed insufficient milk supply (*n* = 8), as a supplement in the absence of perceived or diagnosed insufficient milk supply (*n* = 2), or as part of tradition (*n* = 1). Participants classified as “perceived insufficient milk supply” were women who had not previously been diagnosed with breastfeeding supply issues by a health professional, however they considered themselves as having low milk supply. 

### 3.2. Sources of Recommendation and Supply

Many of the participants had chosen to use herbal galactagogues based on a recommendation from an individual, and in most cases more than one source prompted the decision. Sources of recommendation included advice obtained from friends, family members or other mothers with breastfeeding experience (*n* = 11), midwives (*n* = 5), lactation consultants (*n* = 4), naturopaths (*n* = 4), self-reading and researching (*n* = 4), a child health nurse (*n* = 1), and the Ngala helpline (Ngala is a not-for-profit organization based in Western Australia that provides Early Parenting and Early Childhood services to support and guide families with young children.) (*n* = 1). 

**Table 1:** Summary of the pattern of use: fenugreek as a sole ingredient.

Participants	Product/Dosage Form	Dosage of Administration	Commencement of Therapy (Time Postpartum)	Duration of Therapy
BW 1	Crude seeds cooked with rice as a sweet dessert	Approximately 60 g of seeds cooked with one cup of rice and sugar in pressure cooker, taken twice daily	2 weeks	5.5 months (since 2 weeks postpartum until 6 months—still using)
BW 3	Nature’s Own^®^ Fenugreek capsules *(Each capsule contains 1000 mg fenugreek dry seed powder)*	1 capsule twice daily, then 6 capsules when required as one off dose to boost supply	3 months	9 months (since 3 months postpartum until one year)
BW 6	Nature’s Own^®^ Fenugreek capsules	Initially 1 capsule daily, then increase to 2 capsules daily when required	3.5 months	1 month (since 3.5 months postpartum until 4.5 months—still using)
BW 10	Nature’s Own^®^ Fenugreek capsules	2 capsules three times daily	2 weeks	8.5 months (since 2 weeks postpartum until 9 months)
BW 12	Nature’s Own^®^ Fenugreek capsules	2 capsules in the morning and 2 capsules at night, was aware of the possibility to increase dosage to 6 g daily, however 2 capsules twice daily was sufficient to product an effect.	6 weeks	3.5 months (since 6 weeks postpartum until 5 months—still using)
BW 13	Nature’s Own^®^ Fenugreek capsules	2 capsules three times daily	10 weeks	8 weeks (since 10 weeks postpartum until 18 weeks—still using)
BW 14	Nature’s Own^®^ Fenugreek capsules	Initially 1 capsule daily, then increase to 5 to 6 capsules daily	2 weeks	4.5 months (since 2 weeks postpartum until 5 months—still using)
BW 15	Nature’s Own^®^ Fenugreek capsules	1 capsule three times daily	12 weeks	3 months continuously (since 12 weeks to 6 months), then use when required
BW 18	Nature’s Own^®^ Fenugreek capsules	Initially 1 capsule three times a day for one week, then 2 capsules three times a day	2 months	1 week (ceased therapy due to adverse effect—diarrhoea)
BW 19	Nature’s Own^®^ Fenugreek capsules	2 capsules three times a day	3 months	3 months (since 3 months postpartum until 6 months—still using)

**Table 2:** Summary of the pattern of use: fenugreek and blessed thistle combination products.

Participants	Product/Dosage Form	Dosage of Administration	Commencement of Therapy (Time Postpartum)	Duration of Therapy
BW 2	Nature’s Sunshine^®^ Breast Feeding Support *(Each capsule contains 300 mg fenugreek seed, 150 mg blessed thistle)*	2 capsules twice daily	3 days	8 months (ceased therapy when stopped breastfeeding)
BW 7	Herbs of Gold^®^ Breast-feeding Support *(Each tablet contains 1.5 g fenugreek seed extract, 500 mg blessed thistle)*	1 tablet twice daily	7 months	3.5 months (since 7 months postpartum until 10.5 months—still using)
BW 11	Herbs of Gold^®^ Breast-feeding Support	Initially 5 to 6 tablets daily, then reduce as required	3–4 weeks	18 months (since 3–4 weeks postpartum until 19 months)

**Table 3:** Summary of the pattern of use: naturopaths’ own “lactation tincture” with a combination of herbal ingredients.

Participants	Product/Dosage Form	Dosage of Administration	Commencement of Therapy (Time Postpartum)	Duration of Therapy
BW 4	“Lactation tincture” from naturopath, containing fenugreek and other herbal ingredients which were unknown to participant	1 cup tincture taken four times a day everyday	5 weeks	5 months (since 5 weeks postpartum until 6 months—still using)
BW 5	“Lactation tincture” from naturopath, containing fenugreek and other herbal ingredients which were unknown to participant	5 mL tincture taken six times daily (up to a total daily dose of 30 mL)	9 weeks	7 months (since 9 weeks postpartum until 9 months—still using)
BW 8	“Lactation tincture” from naturopath, containing fenugreek and other herbal ingredients which were unknown to participant	5 mL tincture taken three times daily alternating with twice daily	7 weeks	3 weeks (since 7 weeks postpartum then ceased, restarted at 4 months for 1 week)
BW 9	Weleda^®^ Nursing Tea (Each tea bag contains dry powder of fenugreek seed 500 mg, fennel bitter seed 400 mg, anise seed 400 mg, caraway seed 400 mg, lemon verbena leaves 300 mg)	1 tea bag steeped in hot water, taken once or twice daily	10 weeks	3 months (since 10 weeks until 22 weeks)
BW 16	“Lactation tincture” from naturopath, containing fenugreek and other herbal ingredients which were unknown to participant	5 mL tincture taken once in the morning	3 weeks	14 weeks (since 3 weeks until 17 weeks—still using)
BW 17	“Lactation tincture” from naturopath, containing fenugreek and other herbal ingredients which were unknown to participant	5 mL tincture taken three times daily when required (when breast milk supply was low)	3 months	1 month continuously, then use when required until the time of interview
BW 20	“Lactation tincture” from naturopath, containing fenugreek and other herbal ingredients which were unknown to participant	5 mL tincture taken three times daily or twice daily if missed a dose	6 weeks	2.5 months (since 6 weeks postpartum until 4 months—still using)

Participants were more likely to believe and seek advice from mothers with breastfeeding experience, as illustrated by the following comment:

“…*talking to people on the mothers group page and the breastfeeding support groups, they have all walked through the journey, they understand, and tried different things and suggested different options*.”

(*BW 4*)

The majority of women (*n* = 17) obtained their herbal galactagogues from local community pharmacies. Other sources were health food stores (*n* = 6), naturopathic clinics (*n* = 4) and the local grocery store (*n* = 1).

### 3.3. Perceived Effectiveness and Safety of Herbal Galactagogues

Selected quotations of the perceived effectiveness and safety of herbal galactagogues based on the participants’ personal experience and observation are provided in Table 4, Table 5 and Table 6. Most of the participants (*n* = 16) had “perceived” or “observed” that these herbal galactagogues were effective in promoting breastfeeding adequacy. Some participants commented that they were unable to judge (*n* = 4) due to the simultaneous use of other approaches to increase breast milk supply including use of Motilium^®^ (domperidone) and the “pumping method” through electronic breast pumps.

Various ways to judge or evaluate if a herbal galactagogue was effective or useful were used and referred to “breastfeeding adequacy indicators” for the purpose of this study. The most common indicator was the engorgement of the breasts, with some describing this as the feeling of “fullness”. Other indicators mentioned by participants were changes to the duration of feeding sessions, changes in the infant’s perceived satisfaction and feeding behaviour, the infant’s growth rate or changes in body weight, as well as an increase in the measured volume of expressed milk. Confidence and self-empowerment emerged as an over-arching theme linked to positive experiences with the use of herbal galactagogues. Despite the women not having direct evidence for an increase in milk production, the indicators of breastfeeding adequacy boosted participants’ confidence levels and aided in the delivery of psychological benefits. This enabled participants to experience self-empowerment, which in turn may facilitate exclusive and successful breastfeeding. The attitudes and perspectives of breastfeeding women towards the use of herbal galactagogues in general are reported elsewhere [54]. 

As shown in Table 4, Table 5 and Table 6, the most commonly reported side effects were body odour from the use of fenugreek (*n* = 9), followed by headache (*n* = 2), diarrhoea (*n* = 2), delayed return of menstrual cycle (*n* = 1) and an increase in the number of wet nappies in the infant (*n* = 1), while eight participants stated that no side effect or adverse outcome was observed.

**Table 4:** Perceived effectiveness and safety: fenugreek as a sole ingredient.

Participants	Perceived Effectiveness by User	Adverse Effects Observed
BW 1	Effective, Onset of effect: Within one day	Body odour in mother and infant
	Indicator: Breast engorgement	
	*“So if I have fenugreek tonight, tomorrow morning then I will have very very, what’s the term, engorged breast, a lot of milk, and I found that it was quite instantaneous. Sometimes I even had to express the milk to relieve [the engorgement].The effect was that obvious and instantaneous.”*	
BW 3	Effective, Onset of effect: Within 4–6 h	Sweet-smelling body odour which seemed to be dose-dependent. Odour was absent at 1 or 2 capsules (1–2 g) daily, but present at 6 capsules (6 g) daily.
	Indicators: Breast engorgement, infant’s perceived satisfaction, increased volume of expressed milk	
	*“…I can feel I am full and uncomfortable which normally doesn’t happen till 6am in the morning. Even before feeding, I could feel my breasts so full…it’s just fuller and just quicker feed, the let down is just quicker, there’s more there… I will express 240 mL (before using fenugreek), after using the herb, it will be around 270 mL. At night normally would be 40 mL, but I can express 80 to 90 mL at night after using fenugreek.” “…the duration was shorter, quicker, because at night, it will be 15 min each breast for newborn. Otherwise, it will take 30 min each breast…. When I had it [fenugreek] will just be 15 min each side and she will be quite happy, obviously shorter feeding made her less grumpy. It’s just easier feeding, I think she is fulfilled quicker, the flow was obviously quicker, so it was just like drink, drink, drink, bang!”*	
BW 6	Effective, Onset of effect: Within one day or less than 24 h	None observed
	Indicators: Breast engorgement, infant’s perceived satisfaction and feeding behaviour, infant is putting on w eight	
	*“When my milk comes down, I can see he is really swallowing like a lot, and it will come down more quickly, so it will take less time for all to happen, he feed probably a bit longer sometimes, and that really gulping sort of feeling, seems like a large volume he is getting, compared to before.”*	
BW 10	Unable to judge, was using Motilium^®^, and top-up with infant’s formula	Slight headache
	*“I have no idea. Only because I have limited supply to start with, so I don’t know if it is better or worse since being on it. Same as for Motilium*^®^*, I have been on it since day dot, but I don’t know. They tell me that I have a good supply considering the surgery that I had, but I really don’t know.”*	
BW 12	Effective, Onset of effect: 6–8 h	Slight body odour underarm
	Indicators: Breast engorgement, quicker and more active feeding session, infant’s perceived satisfaction and feeding behaviour, longer interval between each feed	
	*“I could feel that my breasts were very full, and I had to feed her immediately to relief the swelling*.”	
	“*Like if she has had a feed around 4 pm, before that she would cry for a feed about 3 h later, but after taking the tablet, sometimes she can wait till around 8 or 9 before needing to have another feed. I suppose that could be related to her growing up too, you know, when little they tend to feed more, every 2 or 3 h, then slowly become 3 or 4 h.”*	
BW 13	Effective, Onset of effect: 12 h	None observed
	Indicators: Breast engorgement, decreased number of feeds per day	
	“*I have found that since using it myself, that I do feel like my milk supply has been kept quite high because of that*.”	
	“*It has decreased the number of times I feed a day, probably initially because he was getting as much as what was there but it wasn’t enough, so he would be hungry after one or two hours. Whereas now, because he might be getting more at the first feed, he might not feed to feed again after 3 or 4 h.”*	
BW 14	Effective (with higher dose of 6 g daily), Onset of effect: 4–5 h	None at low dose (1 capsule daily), body odour after taking 6 capsules daily
	Indicators: Breast engorgement, ability to express further 50 mL after feeding, quicker and smoother feeding session	
	*“I remember increasing the dosage in the afternoon after lunch and immediately that night I felt that my breasts were so full, they were about to leak I think.”*	
BW 15	Effective, Onset of effect: 2–3 days	None observed
	Indicators: Breast engorgement, increased in volume of expressed milk by 50–100 mL, leaking milk	
	*“I was leaking milk during the night. I would wake up with a wet bra in the morning.”*	
BW 18	Slightly Effective at low dosage (1 tds), however unable to judge for high dosage (6 d) due to adverse effect, Onset of effect: One day	Persistent diarrhoea, therefore ceased usage
	Indicator: Breast engorgement	
	*“Initially with taking three a day, I think it did make some difference, but I would say it still wasn’t enough or ideal. I decided to increase the dosage to six a day. I can’t really tell if taking six a day made any difference because I stopped using it immediately after a day because of diarrhoea and I was too scared to go back to it because now I know that my body doesn’t agree with it. Maybe if my body could tolerate the high dose, it would have helped.”*	
BW 19	Effective, Onset of effect: 1–2 days	None observed
	Indicators: Breast engorgement, infant’s perceived satisfaction and feeding behavior	
	*“I just felt that my breasts were a lot fuller and feeding was quicker and smooth because she isn’t crying. That’s why I am thinking, maybe it is because I have more milk, and she’s getting enough, that’s why she is more satisfied.”*	

**Table 5:** Perceived effectiveness and safety: fenugreek and blessed thistle combination products.

Participants	Perceived Effectiveness by User	Adverse Effects Observed
BW 2	Effective (in maintaining milk supply, not really increasing)	None observed
	Onset of effect: 1 day	
	Indicators: Breast engorgement, infant’s perceived satisfaction	
	*“You could really tell when your breasts are full. Before I kind of always felt, not deflated but you know, if she were longer than usual between feed I wouldn’t really notice it, but then after being on fenugreek, if she went long than say 2 or 2.5 h between feed, it was very obvious, my breast will look very engorged and it will start leaking, which has never happened before.”*	
BW 7	Effective	Sweet-smelling body odour and in urine
	Onset of effect: 2 h	
	Indicators: Breast engorgement, infant’s perceived satisfaction and feeding behaviour, shorter feeding session	
	*“I can see more effective sucking and slurping. After taking fenugreek, it was like suck, swallow, suck, swallow, so she was getting more. It was like proper sucking rather than just getting a bit of milk then just suck for the rest of it. Before that, she would only have proper feed for 10 min then just sucking afterwards, not really swallowing. But after fenugreek, it was like 20 min of constant feeding and swallowing… she seems sort of happier and fuller after feeding. She just appeared to be getting lots of milk and I feel good about that. She appeared more settled.”*	
BW 11	Unable to Judge	None observed
	Onset of effect: 1–2 days	
	Indicators: Breast engorgement, infant’s perceived satisfaction and feeding behavior	
	*“So sometimes the little one will wake up constantly all night just wanting more milk and in the morning still wants more, but whereas from drinking the tea, I kind of feeling my breasts was filled up quicker.”*	

**Table 6:** Perceived effectiveness and safety: naturopaths’ own “lactation tincture” with a combination of herbal ingredients.

Participants	Perceived Effectiveness by User	Adverse Effects Observed
BW 4	Effective, Onset of effect: 4–5 days	Maple-syrupy smell on the body
	Indicators: Infant’s perceived satisfaction and feeding behaviour, increased milk ingested based on baby-weighing method before and after each feed, shorter feeding session	
	*“I still don’t have huge milk supply, but at least I have a milk supply.” “We weighed her, initially the weight was something like 20 g difference, and then after that it got to about 40, 50, 60 g difference. The screaming stopped! She wasn’t getting enough food before, I was getting trouble before sleep, lots of crying and screaming before that. As soon as I started this course of action, my milk increased, she slept better, less screaming, she was getting enough food. Yes, so I wasn’t up until 6am in the morning trying to get her to sleep, put it that way!”*	
BW 5	Effective, Onset of effect: 1–2 days	Delayed return of menstrual cycle, woman believed that it is due to goat’s rue based on her reading
	Indicators: Infant’s perceived satisfaction and feeding behaviour, increased volume of milk supply through supply line	
	*“So for example she might be getting, you know, 5 or 10 mL from me, and 120 mL from the supplement, and then 2 weeks later when I did the milk studies again, she was getting 60 mL from me. Yes. 2 weeks later, and it was 60 mL and sometimes it was up to 90 mL from the breast!” “That definitely helped. I don’t think I would still be able to be breastfeeding if it wasn’t for the herbs. Or, actually, I probably will still be breastfeeding because XXX won’t take a bottle, ever, has never ever taken a bottle. But I haven’t used… I haven’t had to supplement any milk since she was 7 months old. And I think if I wasn’t using the herbs, I would still have to be supplementing.”*	
BW 8	Effective, Onset of effect: Within 24 h	Liquorice-like smell from pores on body, sweat and urine
	Indicators: Breast engorgement, infant’s perceived satisfaction and feeding behaviour, shorter feeding session	
	*“I remember the next morning I woke up, I could just feel immediately that my breasts were really hard and full, and that was like that for a few days, so definitely increase very quickly. I could feel engorged. I know initially the first time I took it I was leaking milk and it was like that for a couple of days. I would say easily 25% increase.” “It made the feeding smoother. Definitely did have an effect on how she is feeding, she was feeding better. Duration of feeding would have been shorter because she was getting milk, more actively feeding throughout the whole time.”*	
BW 9	Effectiveness observed or perceived by user: Unable to judge, Onset of effect: Only slight effect after at least 1 week	None observed
	*“To tell the truth with my supply it didn’t make a huge amount of difference, but I think it did support it slightly.”*	
BW 16	Effective, Onset of effect: 1–2 days	“Sweeter” breast milk
	Indicators: Breast engorgement, infant’s perceived satisfaction and feeding behaviour, increased volume of milk	
	*“I just feel that third time round feeding, my baby is four months old and normally your breasts have settled down and you can’t tell unless it is right before a feed at that four hour mark or whatever, you have a very full breasts, and at the end of the day, they tend to be less full, they felt less full. To me, this time they feel full, there’s something always in there. So I don’t know how to explain that, just feel that there’s always got milk…”*	
BW 17	Unable to judge, believed there’s benefit, so will continue to use, Onset of effect: 3 days	Occasional headaches, mild diarrhoea, slight maple-syrupy smell on body and in breast milk
	Indicators: Breast engorgement, infant’s perceived satisfaction, modest increase in volume of milk expressed	
	*“I can feel my breasts are fuller first of all, and when I express I get a lot more milk. I can see that the baby, because normally before taking it, when I know my milk supply is a bit low, she might need both breasts on a feed. And with this, she would only take one breast for a feed. So there’s definitely sufficient milk in one breast. So that’s how I know my milk supply has gone up again.” “I just feel that the breasts are fuller before a feed…”*	
BW 20	Effective, Onset of effect: Immediate effect, after 1 or 2 days	Slight body odour
	Indicators: Breast engorgement, perceived infant’s satisfaction and feeding behaviour, increase in number of wet nappies	
	*“I could tell because I could feel that they were so full, and I had to breastfeed to relieve the feeling. Also, I think baby was going through a growth spurt at that time, and I was surprise that I had enough milk to feed her, so I was very happy, and I think she is happy too.”*	

## 4. Discussion

This study explored the perspectives on the use, perceived benefits, effectiveness and safety of herbal galactagogues during breastfeeding through interviews with breastfeeding women. The majority of the participants (16 of 20) agreed that the herbal galactagogues of choice were effective in terms of enhancing their breastfeeding adequacy, with fenugreek and blessed thistle the two most commonly used herbal galactagogues, similar to other studies [55,56,57]. Most of the participants (17 of 20) were Caucasian thus minimising ethnic preferences in relation to herbal galactagogues. Fenugreek was used by all 20 participants either as a sole ingredient or in combination with other herbal ingredients. This finding correlates with the results of a population-based survey of 304 breastfeeding women in Perth, Western Australia [58], which indicated that fenugreek was the most popular herbal galactagogue, followed by blessed thistle and fennel. Apart from these, other herbal medicines used as galactagogues by participants were anise seeds, caraway seeds, lemon verbena leaves (as ingredients in the Weleda^®^ Nursing Tea) and possibly other herbal or even non-herbal ingredients used in naturopaths’ own “lactation tincture” unknown to participants. 

As evident in this study, herbal galactagogues exist in various dosage forms and preparations. Although fenugreek was identified as the most commonly used herbal galactagogue, the methods of administration by participants varied: crude seeds, capsules containing dried seed powder, extract tincture and nursing tea. Potency and doses of herbal preparations across different brands were also not standardized, making comparison of effects challenging, as noted by others [55]. In addition, many participants were using relatively low doses of fenugreek, less than the 6 g daily dose (in various forms or preparations) as recommended by the German Commission E [48]. Many commercially available herbal products as seen in this study combined various herbal ingredients in an attempt to maximise galactagogenic effects, which further presents ambiguity when trying to identify the perceived effects of a specific individual herbal galactagogue [56].

Six participants were taking a combination of herbal ingredients as galactagogues in the form of a “lactation tincture” prepared by naturopaths with unknown ingredients, strength or potency, making it impossible to compare products in relation to the effects of specific herbs. Taking into consideration that dosages and length of treatment may influence the efficacy and adverse effect profile, all contents of the products including tinctures prepared extemporaneously should be clearly listed and made available to users. This is important if an emergency health crisis arises where the absence of proper labelling is a potential risk. Besides the variability of dosage and administration, there was also no consistent approach in regards to the commencement and duration of therapy. As evident in this study, women appear to administer herbal galactagogues at various times following birth and for different durations.

Of the six participants who had used “lactation tinctures” obtained from their naturopaths, three (BW 4, BW 8 and BW 20) specifically raised safety concerns with use of medicines when breastfeeding infants and that they believed herbal medicines would be a safer option. Despite expressing concerns over safety issues with the use of medicines whilst breastfeeding, some women continued to use products recommended by their naturopaths, even without any knowledge of the ingredients. 

This in itself is of concern, as participants’ decision to use these products conflicted with their views with regard to safety. It was apparent that these women had inaccurately perceived all herbal medicines in the context as being “natural” and incorrectly thought they will always be “safe” to be used whilst breastfeeding. It is also possible that these women may have built rapport with their naturopaths and that they had trusted their advice and recommendation. It needs to be acknowledged that herbal medicines are not always “safe” and there is a risk that some herbal medicines may cause side effects or potentially toxic effects in both the mothers and their infants if certain constituents are transferred into the breast milk. Furthermore, many herbal medicines lack scientific information to support their efficacy and safety when taken in breastfeeding, as compared to conventional medicines. Some breastfeeding women may have limited knowledge on the risk and benefit profiles of herbal medicines, and the misconceptions surrounding the safety of herbal medicines are of concern. This finding highlights a need to raise the level of public awareness and to provide available information on safety aspects of using herbal medicines, at least amongst breastfeeding women. The scope for improving information dissemination and communication with breastfeeding women on herbal safety issues is hampered by the lack of detailed high level data on this topic. It was clear from this study that breastfeeding women showed high levels of confidence in the safety of herbal galactagogues. This important presumption requires in-depth investigation to elicit the reasons that are informing the confidence and behaviour of breastfeeding women towards the use of herbal medicines.

There were various reasons for use of herbal galactagogues amongst the study population. Women were using herbal galactagogues in both the presence and absence of milk supply issues. More than half (*n* = 12) of the participants were using the herbal galactagogues of choice either due to perceived insufficient milk supply, as a prophylactic supplement or as part of tradition. Although there are physiological or medical reasons for insufficient milk supply, other social and psychological factors may also play an imperative role in affecting the mothers’ milk production [10,56]. Only a minority of the participants sought advice from a lactation consultant or a child health nurse regarding milk supply issues. The perception of inadequacy is common amongst breastfeeding women, leading to anxiety which may affect breastfeeding adequacy and well-being of the women [13]. This indicates a potential psychological role of methods or products used to enhance breastfeeding adequacy. Use of herbal galactagogues as part of self-care during the postpartum period was also observed for some women in this study. As perceived insufficient milk supply, especially during early stages postpartum, was one of the main reasons for commencing herbal galactagogues in this study, the importance of other non-pharmacological measures including education on breastfeeding techniques, encouragement and perseverance should not be neglected. Initiatives to increase women’s awareness of the possibility of various breastfeeding issues that they may encounter including perceived insufficiency and methods to address the issues may assist to avoid early cessation of breastfeeding. Increasing their awareness of the potential issues and the availability of these resources prior to delivery or during the perinatal period may serve to better prepare breastfeeding women for the challenges ahead.

This study reveals that the users of herbal galactagogues were likely to receive advice from and trust their friends and family members who were mothers with breastfeeding experience. Women could relate their personal experiences and emotion to other mothers, hence friends and family members were the most common source of recommendations. Community pharmacies were the main sources of herbal medicines supply including herbal galactagogues which was not unexpected given that community pharmacies are one of the major providers of CMs in the Australian community [59]. 

The adverse effects reported by participants in this study included a maple syrup-like body odour (which was dose-dependent), headache and diarrhoea, which were all consistent with published literature [19,56,57,60]. One participant reported delayed menstrual cycle from the use of a “lactation tincture” supplied by her local naturopath. This “lactation tincture” contained a combination of herbal ingredients including fenugreek and goat’s rue, which she believed to be the cause of this adverse effect. A search of the literature revealed that the hormonal effect experienced by this participant was more likely to be due to fenugreek, as this herbal medicine has been shown to have oestrogenic activity in an *in vitro* study [61].

In the absence of milk volume measurement, women described a range of subjective indicators to “measure” their breastfeeding adequacy. Women in this study described how their choice of therapy was influenced by their perseverance and determination to breastfeed, and their concerns over infants’ safety with the use of conventional treatments. An over-arching theme that emerged was “confidence and self-empowerment”. A sense of autonomy and self-efficacy over their own health needs was recognised as impacting on their level of confidence, at the same time providing women with reassurance throughout the breastfeeding journey and hence having positive psychological effects. Psychological factors may influence the initiation and duration of breastfeeding [62,63]. This is an important finding considering that evidence is lacking to support the use, effectiveness and safety of the majority of herbal galactagogues in breastfeeding. This is in line with findings of a study involving 300 breastfeeding women, using the Breastfeeding Self-Efficacy Scale, which identified that women’s breastfeeding self-efficacy plays a significant role in predicting the duration and success of breastfeeding [64]. Other studies have also supported the need for considering maternal breastfeeding self-efficacy as an important predictor of breastfeeding adequacy [63,65,66,67,68].

This study has highlighted the importance of considering the potential psychological benefits of using herbal galactagogues, and how this translates into breastfeeding adequacy. It should also be noted that qualitative studies involving interviews of herbal galactagogue users or case studies alone are not sufficient to provide evidence to support the clinical efficacy of these medicines. Ideally, a double-blinded randomised controlled trial (RCT) is required to determine the clinical efficacy of these herbal medicines as galactagogues and to determine to what extent, if any, their use could have a placebo effect. 

There appears to be an innate comfort in using herbal medicines with unknown toxicity profiles over a conventional medicine shown to have efficacy and low toxicity in breastfeeding women [69]. Herbal medicines are listed on the Australian Register of Therapeutic Goods (ARTG) by the Therapeutic Goods Administration (TGA) and are issued with AUST L numbers [70]. Herbal medicines are subjected to less rigorous assessments as compared to registered conventional medicines, and are only evaluated for safety and quality, but not their efficacy [71]. In addition, herbal tinctures were used without concern of their contents or toxicity by women who expressed distrust arising from toxicity concerns for conventional medicines. Furthermore, the alcohol content of such a tincture is unknown. This highlights the role that pharmacists could play in educating breastfeeding women to fully comprehend the available (or the lack of) information and the fact that other conventional medicines, such as domperidone, may have higher efficacy and known safety data to support their use in breastfeeding. 

Any research design has its limitations and challenges, and the limitations should be taken into consideration when analysing and interpreting findings of the studies. As with all qualitative research, the background and perspectives of the researchers may have had an impact on the analysis of the interview findings [50]. The interviewer, TFS, is a registered pharmacist in Australia conducting research through a School of Pharmacy, which could have impacted the interviews and analysis. However, to counterbalance the potential bias, regular meetings were scheduled throughout the period of data collection and analysis with the supervisors (LT, LH and JS), who have different disciplinary backgrounds and had no affiliations with any community pharmacies. Participants were recruited through naturopathic clinics and were self-selected, which may not have represented all regular users of herbal galactagogues. Nevertheless, the recruitment methods chosen were considered the most appropriate and met the purpose of the study. 

There are challenges surrounding the pharmacovigilance of herbal medicines in the general population, and more so in breastfeeding women. Further research on the potential and extent of transfer of all or any constituents of a herbal medicine to an infant, will aid in the evaluation of the safety and suitability of these medicines during breastfeeding. 

## 5. Conclusions

The study revealed that women perceived herbal galactagaogues, especially fenugreek, to be effective in enhancing their breastfeeding adequacy. However, despite their long history of use, there are currently limited efficacy and safety data with regard to the use of herbal galactagogues during breastfeeding [56,60]. Most available studies which have examined the clinical efficacy and safety of herbal galactagogues appeared to have small sample sizes and lack consistent approaches in terms of standardizing study protocols and processes [55]. Although the qualitative nature and sample size of this study does not allow for a definitive conclusion as to which type(s) of herbal galactagogue(s) were effective or safe, the exploratory nature of the study delivered better understanding of the topic in the Australian context and provided direction to subsequent research in the field. The findings of this study, along with a review of the relevant literature, identified a need for scientific evaluation of the commonly used herbal galactagogues in Australia, namely fenugreek and blessed thistle. 
